# A 3D Bioprinting Approach to Studying Retinal Müller Cells

**DOI:** 10.3390/genes15111414

**Published:** 2024-10-31

**Authors:** Davide Vecchiotti, Mauro Di Vito Nolfi, Francesca Veglianti, Francesca Dall’Aglio, Hafiz Nadeem Khan, Irene Flati, Daniela Verzella, Daria Capece, Edoardo Alesse, Adriano Angelucci, Francesca Zazzeroni

**Affiliations:** 1 Department of Biotechnological and Applied Clinical Sciences (DISCAB), University of L’Aquila, 67100 L’Aquila, Italy; 2 Department of Experimental Medicine, Sapienza University of Rome, 00161 Rome, Italy

**Keywords:** bioprinting, 3D culture, rMC-1 cells, Müller cells, retinal diseases

## Abstract

**Background/Objectives:** Bioprinting is an innovative technology in tissue engineering, enabling the creation of complex biological structures. This study aims to develop a three-dimensional (3D) bioprinted model of Müller cells (MCs) to enhance our understanding of their physiological and pathological roles in the retina. **Methods**: We investigated two different hydrogels for their ability to support the viability and differentiation of rMC-1 cells, an immortalized retinal cell line. Using 3D bioprinting technology, we assessed cell viability, differentiation, and functional characteristics through various assays, including live/dead assays and western blot analysis. **Results**: The collagen-based hydrogel significantly improved the viability of rMC-1 cells and facilitated the formation of spheroid aggregates, more accurately mimicking in vivo conditions compared to traditional two-dimensional (2D) culture systems. Moreover, 3D bioprinted MCs exhibited reduced markers of gliosis and oxidative stress compared to 2D cultures. Molecular analysis revealed decreased expression of GFAP and phosphorylated ERK in the 3D setting, indicating a less stressed cellular phenotype. **Conclusions**: Our findings demonstrate that 3D bioprinting technologies provide a more predictive platform for studying the biology of retinal MCs, which can help in the development of targeted therapeutic strategies for retinal diseases.

## 1. Introduction

Bioprinting is a promising technology that has gained worldwide attention for its application in various fields, such as tissue engineering, regenerative medicine, drug screening, and high-throughput assays. 3D bioprinting or additive manufacturing (AM) involves the simultaneous printing of living cells, growth and differentiation factors, and biomaterials with a prescribed layer-by-layer stacking organization using computer-aided design (CAD) or computer-aided manufacturing (CAM) [1,2]. The development of numerous bioprintable materials has allowed scientists to manipulate the biological and biochemical environments to create a complex biological construct with living cells [1,3]. Unlike 3D printing, 3D Bioprinting uses biomaterials also called bioinks to mimic the complex structures of biological materials. Accordingly, bioinks should be printable and cytocompatible and should not affect the shape, mechanical stability, cell viability, proliferation, differentiation, migration, or subsequent tissue formation. The selection of bioinks depends on both the type of bioprinter and the bioprinting approach used to construct tissue-like 3D structures [4]. Although a variety of somatic cells have been used in 3D bioprinting, most applications rely on the use of immortalized cell lines and stem cells to facilitate de novo tissue development [4,5,6]. In the human retina, Müller cells (MCs) are the principal glial cells. They are located not far from almost all retinal components, such as photoreceptors, secondary neurons, ganglion cells, vasculature, and vitreous, supporting the entire retinal structure and function [7,8,9,10]. It is recognized that MCs play a pivotal role in both the physiological and pathological processes of the retina. Due to their unique position and connections within the retina, MCs control several functions, including the regulation of the structural stability and metabolic homeostasis of the retina and the differentiation of other cells after damage [11,12,13,14]. Accordingly, in pathological retinal conditions, including glaucoma, MCs show pathological features that trigger cytotoxicity, oxidative stress, and neuroinflammation [15,16]. Herein, understanding the molecular biology of MCs and their interaction with other cells underpinning the MC-mediated biological functions could enhance our knowledge of the pathophysiological changes associated with retinal diseases, ultimately improving patient prognosis. However, studying the biological responses of MCs using traditional 2D culture systems has many limitations [17,18], as they fail to reproduce the specific cellular structure of the retina and the stereographical characteristics of MCs in the retinal tissue. In this research, we aimed to develop MC-based 3D structures that could mimic MCs’ in vivo physiological characteristics. Two different hydrogels have been tested to identify specific trophic support in which MCs can thrive and grow. Finally, we compare this advanced technology with the classical 2D culture, demonstrating the usefulness of the 3D bioprinting model to study the complexity of retinal MCs.

## 2. Materials and Methods

### 2.1. Cell Culture and Post-Printing Viability

Rat retinal Müller cells (rMC-1) were cultured in DMEM F12 (Euroclone, cat. N. ECM0095L, Milan, Italy) supplemented with 10% Fetal Bovine Serum (FBS) (Gibco, cat. N. A5256801, Thermo Fisher Scientific, Wilmington, NC, USA), 1% penicillin/streptomycin (100 U/mL; 100 µg/mL) (Euroclone, cat. N. ECB3001D, Milan, Italy), and 1% L-glutamine (2 mM) (Euroclone, cat. N. ECB3000D, Milan, Italy) at 37 °C with 5% CO_2_ and were maintained for seven days both in 2D and in 3D conditions. Post-printing viability of 3D cultures was assessed 24 h after printing using PrestoBlue™ Cell Viability Reagent (Invitrogen cat. N. A13262, Thermo Fisher Scientific, Wilmington, NC, USA) following the manufacturer’s instructions and recorded by spectrophotometric analysis (µ-Quant plate-reader, Bio-Tek Instruments, Winooski, VT, USA).

### 2.2. Sodium Alginate–Gelatin-Based Bioinks Preparation

Two different hydrogels were used for bioprinting: Alginate 2%/Gelatin 8% (BI1) and Alginate 2%/Gelatin 8%/Collagen100 µg/mL (BI2). Briefly, UV-sterilized Sodium Alginate (SA) (Sigma Aldrich cat. N. W201502, Merck group, St. Louis, MO, USA) and Gelatin Porcine Skin Type A (GEL) (Sigma Aldrich cat. N. G2500, Merck group, St. Louis, MO, USA) powders were dissolved in sterile phosphate buffer saline (PBS) (Euroclone cat. N. ECB 4004, Milan, Italy) at the above-mentioned concentrations under a laminar flow hood. To facilitate the process, the solution was kept at 50 °C with continuous stirring on a hotplate. Once dissolved, an aliquot of the SA-GEL was supplemented with Bovine Collagen Type I (Sigma Aldrich cat. N. C4243, Merck group, St. Louis, MO, USA) at a final concentration of 100 µg/mL. The two formulations were then centrifugated to remove any bubbles, dispensed in sterile luer-lock syringes, and stored at 4 °C for further use.

### 2.3. Bioprinting Process

The structure, wide 20 mm and high 0.7 mm, was designed with PrusaSlicer software (version V.2.7.1). Before bioprinting, rMC-1 cells were harvested with Trypsin-EDTA solution (Euroclone cat. N. ECB3052D, Milan, Italy), centrifugated at 1200 rpm, and counted with a Corning Cell Counter (Corning, NY, USA). A total of 10.5 × 10^6^ cells were collected, resuspended in 300 µL of fresh medium, and loaded in a 5 mL luer-lock syringe. A second 5 mL syringe was loaded with 2.7 mL of prewarmed hydrogel. The two syringes were then connected to mix the contents and generate a cell-laden hydrogel at a final concentration of 3.5 × 10^6^ cells/mL. Encapsulated cells were then transferred to the bioprint cartridge, which was subsequently transferred to the temperature-controlled printhead of the Cellink BIO X 3D Bioprinter (Cellink, Gothenburg, Sweden). For optimal extrusion, the temperature of the printhead and the print bed were set at 29 °C and 25 °C, respectively, and the extrusion was carried out in a 6-well plate at a pressure of 55 kPa, at a speed of 5 mm/s and with a 22 gauge needle. Finally, bioprinted structures were crosslinked for 10 min in CaCl_2_ at 100 mM (Sigma Aldrich, cat. N. C7902, Merck group, St. Louis, MO, USA) and for a further 2 min in BaCl_2_ (Sigma Aldrich, cat. N. 217565, Merck group, St. Louis, MO, USA), washed in PBS, and cultured in fresh medium at standard conditions.

### 2.4. Morphological Analysis and Imaging

The morphology and viability of 3D printed structures over time were assessed by ActinGreen™ 488 ReadyProbes™ Reagent (Invitrogen, cat. N. R37110, Thermo Fisher Scientific, Wilmington, NC, USA) and LIVE/DEAD Cell Viability Assay (Invitrogen cat. N. R37601, Thermo Fisher Scientific, Wilmington, NC, USA), respectively, following the manufacturer’s instructions. Images were acquired by an AxioZoom V16 microscope (Zeiss, San Diego, CA, USA) coupled with ZEN Blue 3.3 Pro software and a confocal microscope Leica TCS SP5 II. Images showing the 3D spatial distribution of rMC-1 cells are qualitative visualizations where the pseudocolor is not linked to the xy intensity but encodes the z-position of the section. The coded sections were then superimposed. Quantification and size distribution analysis were performed using Fiji software, version 2.9.0.

### 2.5. Protein Extraction and Western Blot Analysis

For total protein extraction, 2D cells were washed in PBS and lysed in RIPA buffer using a cell scraper. Cell extracts were sonicated, centrifugated at 10,000 rcf at 4 °C, and stored at −80 °C. For 3D cell cultures, the matrix was dissolved using a 250 mM EDTA solution in PBS and centrifuged at 400 rcf at 4 °C. Cell pellets were then washed with PBS and processed as described previously. Nuclear and cytoplasmic extracts were obtained by processing cell pellets following our subcellular fractionation protocol reported in [19]. Protein concentration was assessed with the Pierce™ BCA Protein Assay Kit (Thermo Scientific, cat. N. A55865, Thermo Fisher Scientific, Wilmington, NC, USA), and an equal amount of proteins was resolved on polyacrylamide gel, transferred onto nitrocellulose membranes, blocked with 10% non-fat dry milk, and incubated overnight with the following antibodies: Vimentin 1:5000 (Cell Signaling, cat. N. 5741, Danvers, MA, USA), pSTAT3 Y705 1:1000 (Cell Signaling, cat. N. 9131, Danvers, MA, USA), STAT3 1:3000 (Cell Signaling, cat. N. 9139), pERK 1:10,000 (Cell Signaling, cat. N. 9101), ERK 1:10,000 (Cell Signaling, cat. N. 9102), SOD1 1:5000 (Cell Signaling, cat. N. 37385, Danvers, MA, USA), DJ1 1:5000 (Santa Cruz Biotechnology, cat. N. sc-55572, Dallas, TX, USA), Thioredoxin 1 1:1000 (Cell Signaling, cat. N. 2429, Danvers, MA, USA), β-Actin 1:4000 (Santa Cruz Biotechnology, cat. N. sc-1615), PCNA 1:10,000 (Cell Signaling, cat. N. 13110, Danvers, MA, USA), GFAP 1:500 (Cell Signaling, 3670, Danvers, MA, USA), TNFα 1:2000 (Abcam, cat. N. AB6671), IL6 1:6000 (Abcam, cat. N. AB9324, Cambridge, UK), pp70S6k 1:500 (Cell Signaling, cat. N. 9205, Danvers, MA, USA), pAMPKα 1:1000 (Cell Signaling, cat. N. 2535), pAkt T308 1:1000 (Cell Signaling, cat. N. 13038, Danvers, MA, USA), pAkt S473 1:2000 (Cell Signaling, cat. N. 4060, Danvers, MA, USA), GLUL (D2O3F) 1:1000 (Cell Signaling, cat. N. 80636, Danvers, MA, USA), lamin A/C 1:20,000 (Santa Cruz Biotechnology, cat. N. sc-376248, Dallas, TX, USA), α-Tubulin 1:15,000 (Cell Signaling, cat. N. 2144, Danvers, MA, USA). Densitometric analysis of immunoblotting was performed as reported in [20].

## 3. Results

### 3.1. Collagen-Based Bioink Supports rMC-1 Cells Viability

**Figure 1:**
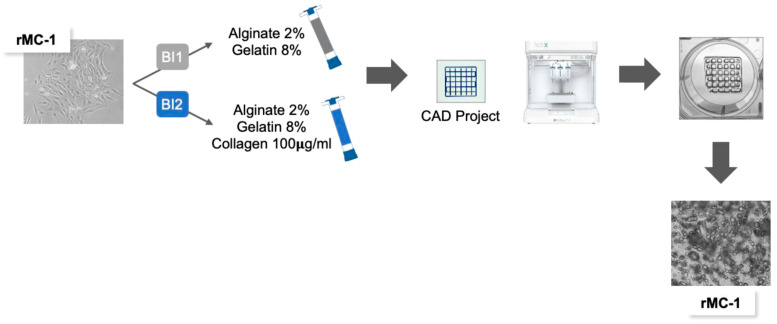
Schematic representation of the bioprinting procedure used in the study. Three-dimensional cultures were obtained using rMC-1 cells and two different hydrogels (BI1, grey syringe: Alginate 2% + Gelatin 8%; BI2, blue syringe: Alginate 2% + Gelatin 8% + Collagen 100 μg/mL).

**Figure 2:**
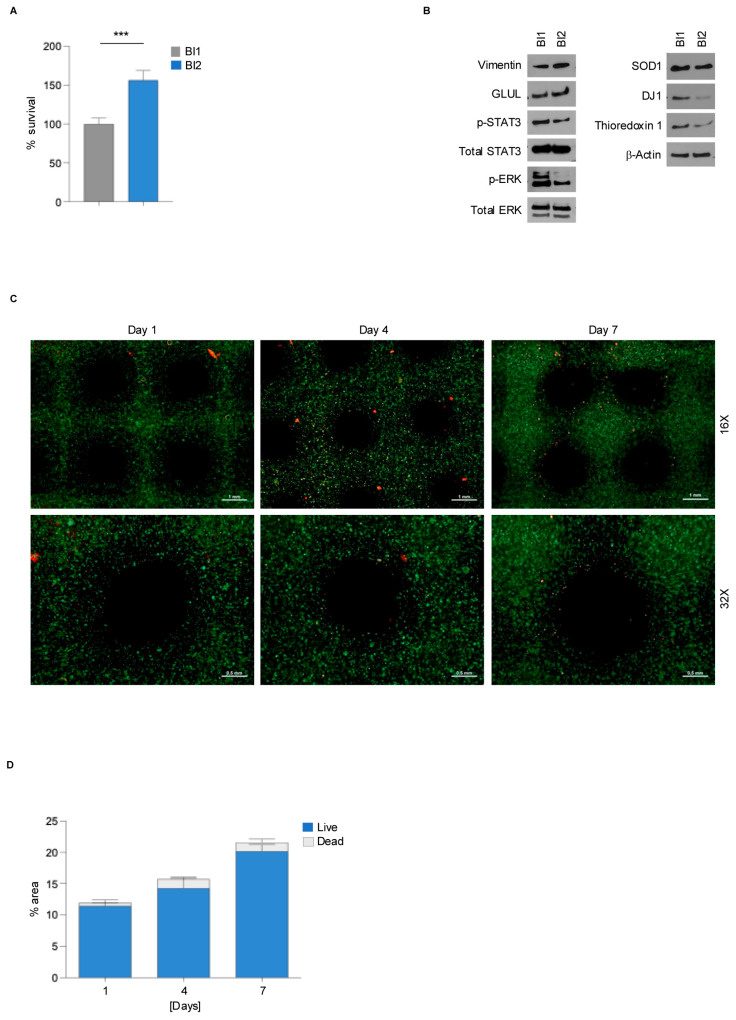
Viability assay of rMC-1 cells bioprinted with different hydrogels. (**A**) PrestoBlue assay showing the percentage of viable rMC-1 cells bioprinted with BI1 or BI2 one day post-printing. Values denote mean ± SD. Statistical significance was calculated by a 2-tailed Student’s *t*-test. *** *p* < 0.001. (**B**) Western blot showing the expression levels of gliosis markers (p-STAT3, Total STAT3, p-ERK, Total ERK), differentiation markers (Vimentin, GLUL), and oxidative stress markers (SOD1, DJ1, and Thioredoxin 1) in rMC-1 cells bioprinted for seven days as described in (**A**). β-Actin is shown as the loading control. (**C**) Fluorescence microscopy images from the live/dead assay showing the live (green) and dead (red) cells bioprinted with BI2 at the indicated time points. Scale and magnification are shown. Magnification 16×, scale = 0.5 mm; magnification 32×, scale = 1 mm. (**D**) Histogram showing the percentage of the area covered by live or dead rMC-1 cells bioprinted as shown in (**C**).

rMC-1 is an immortalized cell line obtained from adult rat retina, widely used in molecular ophthalmology due to its ability to recapitulate the phenotype of primary Müller cells [21,22]. To better characterize the molecular biology of rMC-1 cells in a more reliable culturing setting, two alginate-based bioinks were prepared to set up a 3D model of MCs: Alginate 2%/Gelatin 8% (BI1) and Alginate 2%/Gelatin 8%/Collagen 100 µg/mL (BI2). Using 3D bioprinting technology (BioX, Cell-INK), CAD-designed rMC-1 structures were printed using both BI1 and BI2 hydrogels (Figure 1). To assess whether cell viability was influenced by the bioink composition, a Presto Blue viability assay was performed one day post-printing. rMC-1 cells bioprinted with BI2, containing collagen, showed increased viability compared to BI1, suggesting that collagen is required for rMC-1 cell-laden bioink, as they provide better support for their viability (Figure 2A). Given the recognized structural and antioxidant functionalities of MCs in the retina microenvironment, both under normal and pathological conditions [23], we investigated the differentiation, gliosis, and redox status of rMC-1 cells printed with the two different bioinks using western blot analysis (Figure 2B and Figure S1A). Compared to BI1, the collagen-based bioink induced an increase in the MC differentiation markers Vimentin and Glutamine Synthetase (GLUL), coupled with a significant decrease in gliosis markers (pSTAT3, p-ERK) and anti-oxidative stress markers (SOD1, DJ1, Thioredoxin 1). The biocompatibility and ability of BI2 to sustain the vitality of rMC-1 cells have been further confirmed by a live/dead assay that showed a very high ratio of viable cells (Figure 2C,D and Figure S1B–G) at days 1, 4, and 7 post-printing. Overall, these results suggest the advantages of using collagen-based hydrogel for 3D bioprinting processes to achieve improved cell viability and more reliable results.

### 3.2. Spatially Defined Pattern of 3D Bioprinted rMC-1 Cells Shows Heterogeneous Spatial Distribution and Tendency to Form Spheroid Structures

**Figure 3:**
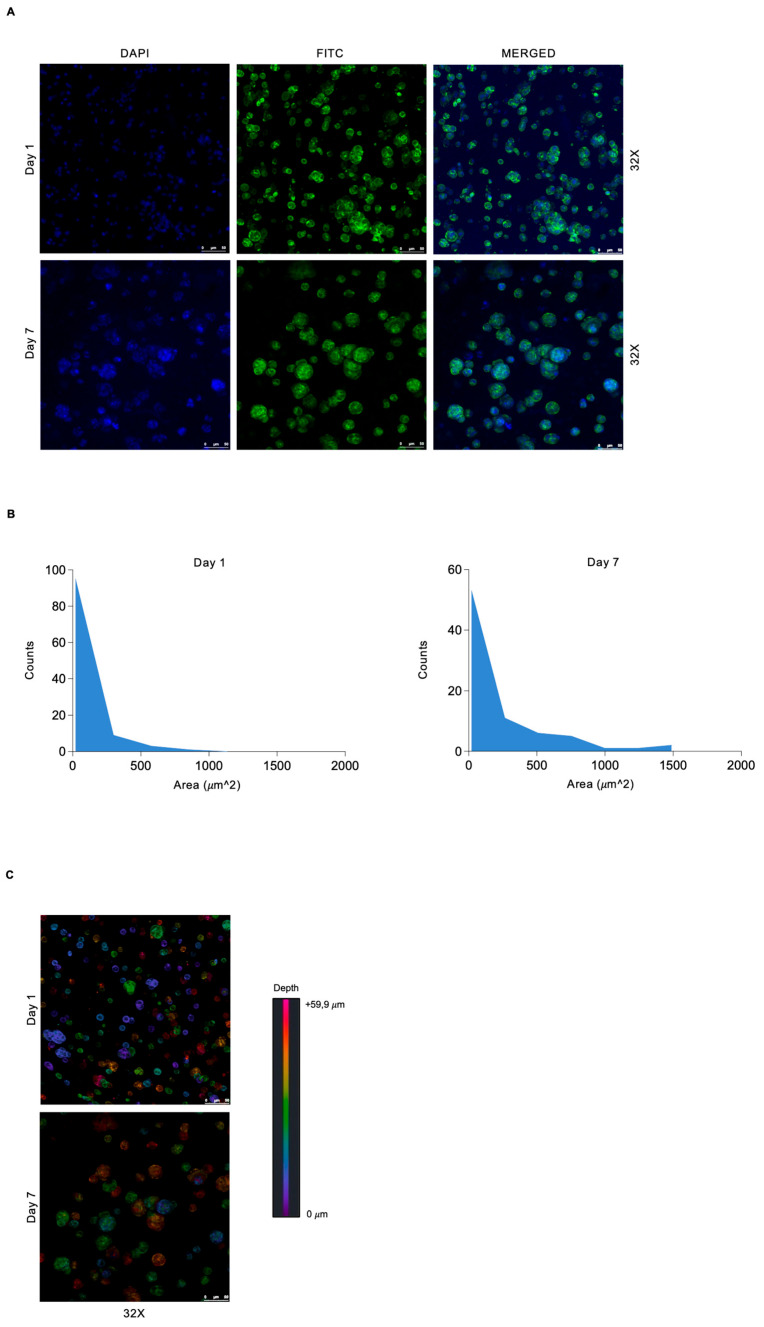
3D bioprinted rMC-1 cells aggregate into spheroid structures. (**A**) Fluorescence staining of rMC-1 cells bioprinted with BI2 at the indicated time points. DAPI (blue) stains nuclei, and phalloidin (green) stains actin. Scale (50 μm) and magnification are shown. (**B**) Distribution of rMC-1 spheroid areas expressed as square inches from the experiment in (**A**). (**C**) Images showing the 3D spatial distribution of rMC-1 cells bioprinted as shown in (**A**) on days 1 and 7. Scale (50 μm) and magnification are shown.

After identifying the best suitable hydrogel capable of supporting rMC-1 cell growth and function in a 3D environment, we further investigated their distribution within the chosen 3D bioprinted SA-GEL-collagen-based hydrogel. Cytocompatibility and spatial distribution were confirmed by IF staining for phalloidin-F-Actin, showing cell morphology of 3D bioprinted cell-laden matrices on day 1 and day 7 (Figure 3A). The formation of spheroid cell structures was confirmed at both time points, with a marked increase in the spheroid size and distribution on day 7 (Figure 3B). Confocal microscopy analysis showed a homogeneous distribution across the matrix, confirming the quality and fidelity of the bioprinting process (Figure 3C). Consistent with the widely documented relationship between cell survival/cell morphology and shear stress during the bioprinting process [24], our results suggest that SA-GEL-collagen-based hydrogel supports the tendency of MCs to aggregate in a spheroid-like manner.

### 3.3. 3D Bioprinted Müller Cells Show Reduced Gliosis and Oxidative Stress Compared to Conventional 2D Culture

**Figure 4:**
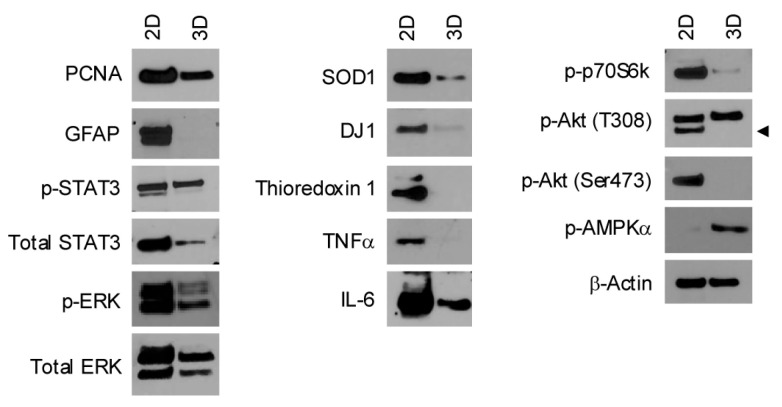
rMC-1 cells bioprinted with Alginate 2% + Gelatin 8% + Collagen 100 μg/mL show a reduction of gliosis and oxidative stress markers. Western blot showing the expression levels of proliferation (PCNA), gliosis (GFAP, p-STAT3, Total STAT3, p-ERK, Total ERK), oxidative stress (SOD1, DJ1, Thioredoxin 1), inflammatory (TNFα, IL-6), and metabolic (p-p70S6k, p-Akt (T308), p-Akt (Ser473), p-AMPKα) markers in 3D bioprinted rMC-1 cells versus 2D cultured rMC-1 cells after 7 days of culture. β-Actin is shown as the loading control arrow indicates the p-Akt (T308) protein.

Neuroinflammation and oxidative stress are key processes occurring in the pathological retinal microenvironment [25,26,27]. Therefore, we evaluated whether our 3D model of bioprinted MCs may provide a more accurate representation of these processes compared to conventional 2D cultures. Our data demonstrated that MCs bioprinted in collagen-based bioink and cultured for seven days showed a reduction of both proliferation (PCNA) and gliosis markers (pSTAT3, pERK, and GFAP) compared to the bidimensional MC culture (Figure 4). Moreover, to further confirm the activity of STAT3 and ERK transcription factors, we performed a subcellular fractionation to selectively extract cytoplasmic and nuclear proteins. As expected, STAT3 and ERK are more active in 2D cells, as confirmed by the increased presence of pERK and pSTAT3 in the nuclear fraction (Supplementary Figure S2A). In addition, a significant reduction of both antioxidative markers (SOD1, DJ1, and Thioredoxin 1) and proinflammatory cytokines (TNFα and IL-6) was observed in 3D bioprinting MCs compared to 2D cultures. Furthermore, 2D and 3D MC cultures exhibited different metabolic profiles, with 3D bioprinted MCs showing a reduction in mTOR signaling (pospho-p70S6k) and AKT (pAktT308 and pAkt Ser 473) and an upregulation of AMPK (pAMPKα) expression (Figure 4 and Figure S2B). Overall, these results demonstrate that, unlike conventional 2D cultures, the developed 3D bioprinting model more accurately reflects the molecular biology of non-activated MCs, making it a more suitable model for studying MC biology under both physiological and pathological conditions.

## 4. Discussion

Although two-dimensional (2D) cell cultures have played a pivotal role in research, their limitations are now widely recognized. In light of this, three-dimensional (3D) cell culture has become essential for advancing our understanding of cellular behavior. Hydrogel-based bioprinting techniques are unique due to their ability to mimic the ECM while allowing soluble factors such as cytokines and growth factors to travel through the tissue-like gel [28]. Compared to traditional 2D cell culture systems, 3D cultures better simulate cell-to-cell interactions in a complex microenvironment, such as that of the retina, providing more reliable and accurate data, similar to what is observed in in vivo studies [29]. Although other valuable techniques were employed to mimic the layered structure of the retina [30,31], we preferred an extrusion-based and scaffold-free approach which prioritizes the biological behavior of Müller cells in 3D. Future work could combine extrusion-based methods with higher-resolution techniques to create models that better mimic retinal layering while supporting Müller cell functionality in 3D.

The findings reported in this study further emphasize the importance of using an appropriate culturing method that resembles the in vivo conditions of Müller cells within the retina. We developed and tested two different hydrogels able to support Müller cell growth in vitro, and we demonstrated that collagen is crucial for supporting the viability and stereodistribution of rMC-1 cells. These findings are in agreement with the widely accepted roles that extracellular matrix (ECM) plays in Müller glia activation and subsequent gliotic processes [32]. Hence, collagens are the main components of the ECM of most soft tissues, including the retina, where they form a fibrillar net in charge of maintaining its structural strength, attachment to the vitreous, and the retinal vasculature [33]. Collagen not only maintains the mechanical elasticity of the tissue but also provides support for the life activities of cells. It is perceived as an endogenous component of the human body and displays unique intrinsic properties (e.g., cell recognition signals, ability to form 3D scaffolds of various physical conformations, controllable mechanical properties, and biodegradability, etc.), which make it an excellent choice for the raw material of tissue regeneration [34,35,36]. Furthermore, consistent with previous in vitro and in vivo data where isolated MCs demonstrated the ability to produce clonal neurospheres, our data demonstrated that bioprinted MCs form spheroidal aggregates. This suggests a potential for further investigation into their self-renewing and multipotent capabilities, as well as their intriguing potential to differentiate into functional neurons [37,38].

We further demonstrated that rMC-1 cells when bioprinted in collagen-based bioink are less prone to gliosis and oxidative stress compared to those cultured under classical 2D conditions, supporting the need to move beyond traditional in vitro approaches toward more complex models. Hence, our results, based on the expression of widely used biomarkers, confirmed significant molecular differences between Müller cells cultured in 2D and those bioprinted in 3D. In particular, we demonstrated that compared with 2D cultures, rMC-1 3D cells do not express detectable levels of GFAP, a marker associated with tissue stress and retinal damage [39,40]. This result is further supported by the reduced expression of STAT3, which is widely recognized as a driver of GFAP expression in several neurodegenerative diseases [41,42,43]. 3D-cultured rMC-1 cells also showed reduced levels of phosphorylated ERK. The activation of MAPK (ERK) signaling, predominantly in Müller cells, is a recognized response to gliosis and cellular stress [26,44,45]. Zeng et al. demonstrated that p-ERK is activated mainly in Müller cells under stress in ex vivo human retinal explants, in murine retinas damaged by photo-oxidation, and in a human eye with late-stage GA (Geographic Atrophy), supporting previous reports of ERK activation in glial cells in diseased animals [44,46,47].

Overall, our findings suggest that activation of pERK in Müller cells observed in 2D, but not in 3D settings, could be induced in response to stressful culturing conditions. Oxidative stress and neuroinflammation have recently been associated with several retina dysfunctions. In this context, MCs have been recognized as crucial players in maintaining homeostatic conditions when a noxa occurs [48,49]. Compared to 2D cells, 3D cultures showed a decreased level of antioxidative stress markers SOD1, DJ1, and thioredoxin1. Moreover, given the crucial role of autophagic flux cells in response to oxidative stress in the retina and in Müller cells [50,51], we also evaluated the AMPK-mTOR axis as a master regulator of this process [52]. PI3K-Akt signaling is also involved in response to oxidative stress stimuli [53]. We reported that while MCs in 2D culture show a strong activation of PI3K-Akt-mTOR signaling, suggesting a basal activation of the antioxidant defense system in standard culture conditions, 3D cells switch off the PI3K-Akt-mTOR pathway and show a strong AMPK activation, suggesting a less prone phenotype to oxidative stress under basal conditions [51]. Interestingly, since AMPK and mTOR regulate catabolic and anabolic reactions, respectively [54], these diametrically opposed molecular profiles likely reflect specific metabolic adaptations to different extracellular conditions, highlighting how the extracellular environment can strongly affect cellular molecular biology and subsequent in vitro studies.

Thus, our 3D model of MCs effectively reduced oxidative stress and expression of proinflammatory cytokines, suggesting the suitability of bioprinting technologies to study pathophysiologic in vitro retinal models while avoiding the basal expression of a reactive phenotype in MCs. This bioprinted model will be further improved in order to evaluate the existence of cell–cell interactions, thus providing a more predictive retinal model. Although significant efforts must be made to optimize cell–matrix and cell–cell interactions to better mirror the tissue in vitro, bioprinting techniques have the potential to represent a step forward in the reconstruction of the physiologic cell phenotype.

## 5. Conclusions

In summary, this study highlights the potential of 3D bioprinting technology to create more physiologically relevant models of retinal Müller cells (MCs) compared to traditional 2D models, which have limitations in capturing the complex molecular and structural biology of MCs. By more accurately mimicking the native retinal environment, the 3D bioprinted model offers a promising platform for studying MC biology, contributing to a better understanding of retinal pathophysiology and advancing the development of therapeutic interventions. Future work can build upon this model by enhancing cell–cell interactions and refining extracellular matrix components, further bridging the gap between in vitro and in vivo models for retinal research.

## Data Availability

The original contributions presented in the study are included in the article/Supplementary Materials, further inquiries can be directed to the corresponding author.

## Supplementary Materials

The following supporting information can be downloaded at: https://www.mdpi.com/article/10.3390/genes15111414/s1, Supplementary Figure S1. (A) Densitometric analysis of western blot showed in Figure 2 showing the expression levels of gliosis markers (p-STAT3 Y705, p-ERK) differentiation markers (Vimentin, GLUL) and oxidative stress markers (SOD1, DJ1 and Thioredoxin 1). (B) Fluorescence microscopy images from the Live/dead assay showing the live (green) rMC-1 cells bioprinted with BI2 Alginate 2% + Gelatin 8% + Collagen 100 mg/mL at the indicated time point. (C) Fluorescence microscopy images from the Live/dead assay showing the dead (red) cells bioprinted as in B. (D) Fluorescence mi-croscopy images from the Live/dead assay showing the live (green) and dead (red) cells bioprinted with BI1 Algi-nate 2% + Gelatin 8% at the indicated time points. (E) Histogram showing the % of area covered by live or dead rMC-1 cells bioprinted as in D. (F) Fluorescence microscopy images from the Live/dead assay showing the live (green) rMC-1 cells bioprinted as in D. (G) Fluorescence microscopy images from the Live/dead assay showing the dead (red) cells bioprinted as in D. Scale and magnification are shown. Magnification 16×, scale = 1mm; magnifica-tion 32×, scale = 0.5 mm. Supplementary Figure S2. (A) Western blot analysis of cytoplasmic (C) and nuclear (N) fractions of p-STAT3 and p-ERK from 3D bioprinted rMC-1 cells or 2D cultured rMC-1 cells after 7 days. Tubulin and Lamin A/C were used as fractionation controls. (B) Densitometric analysis of western blot showed in Figure 4.
