# Supplementary figures and images for: A 3D Bioprinting Approach to Studying Retinal Müller Cells
Source: Genes (Basel). 2024 Oct 31;15(11):1414. doi: 10.3390/genes15111414 (PMC11593586; doi:10.3390/genes15111414)

Supplementary Figure S1

A

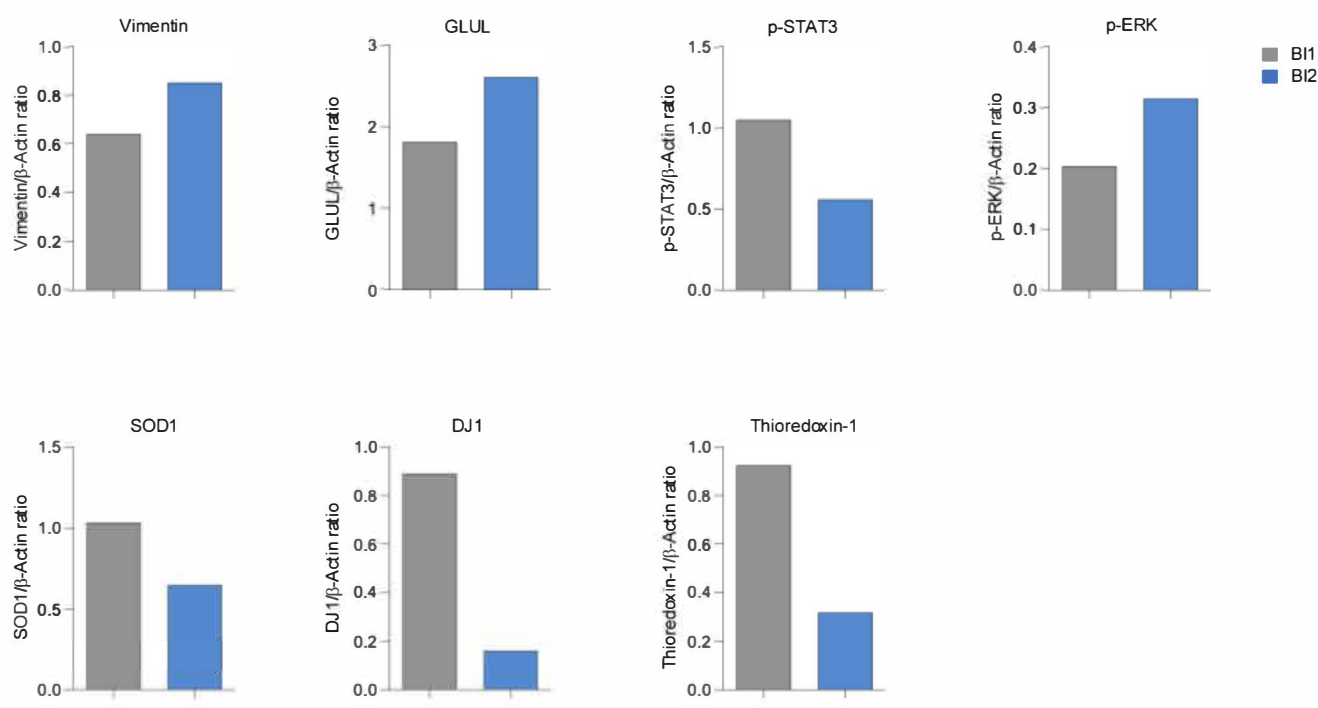

B

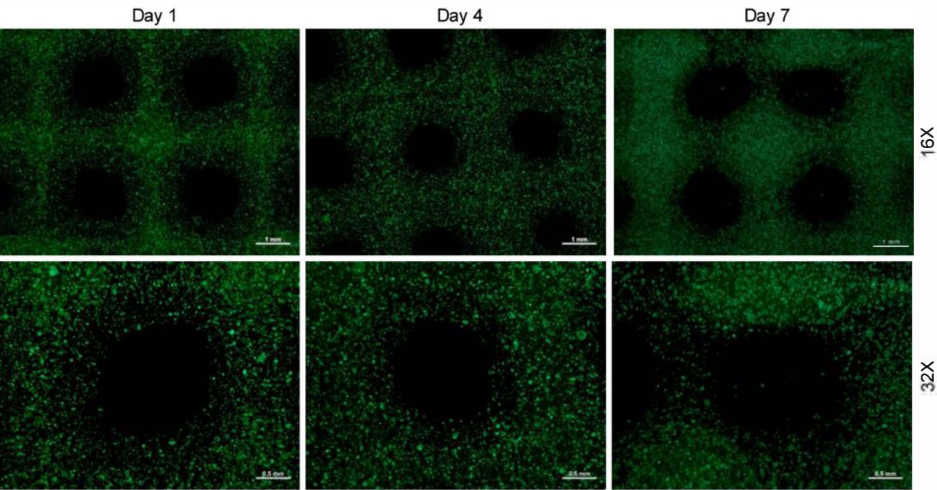

C

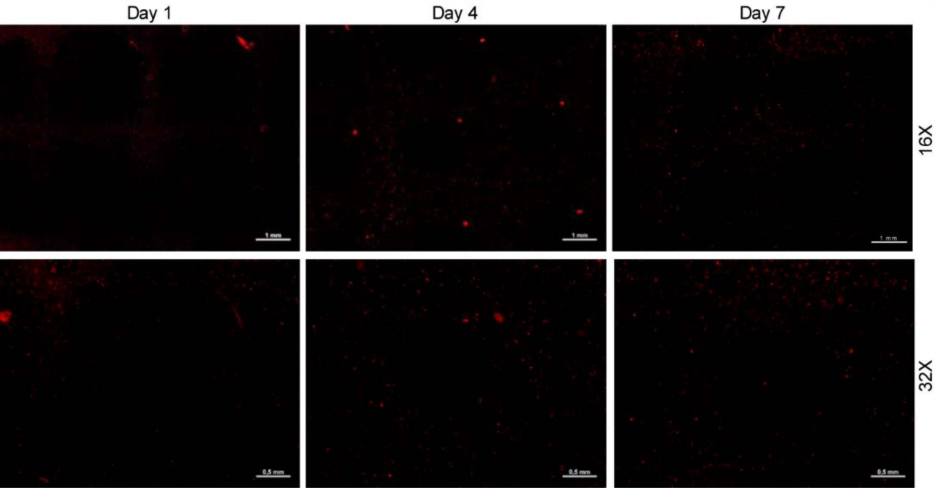

D

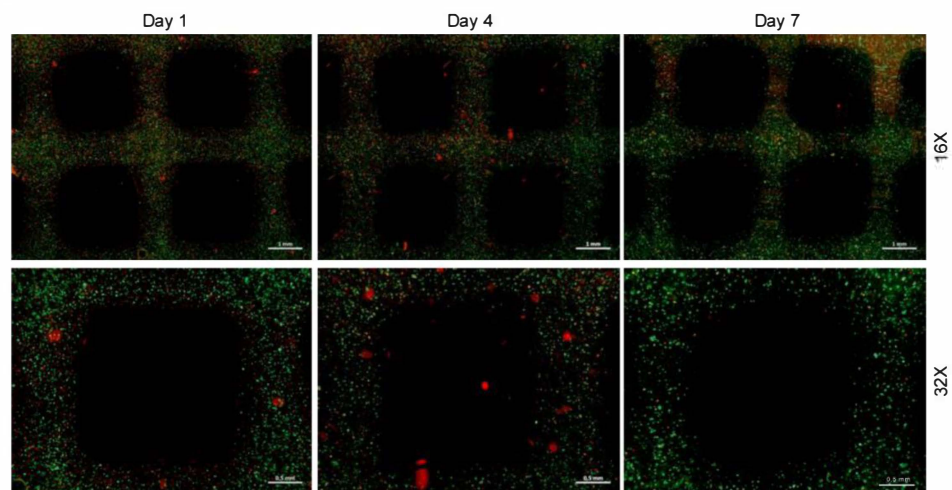

E

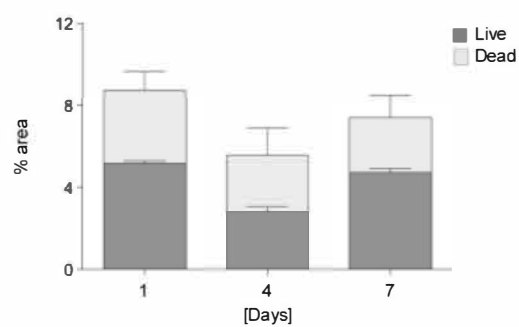

F

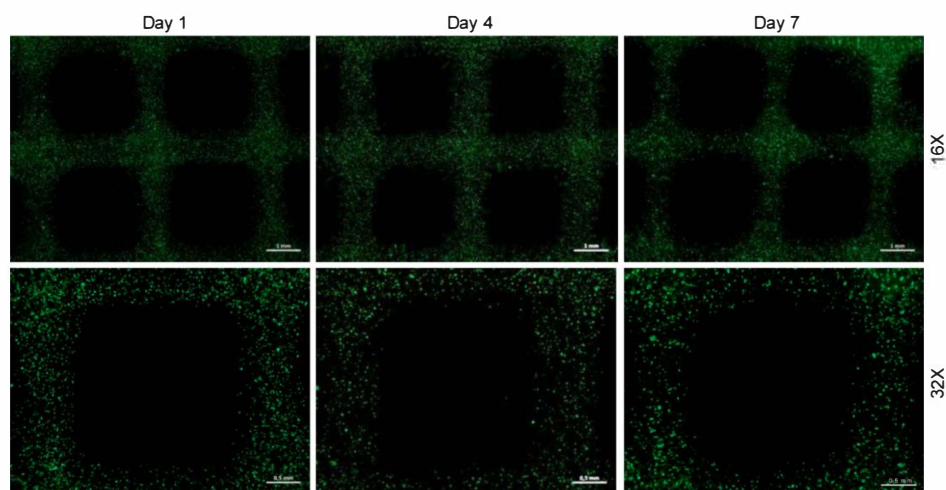

G

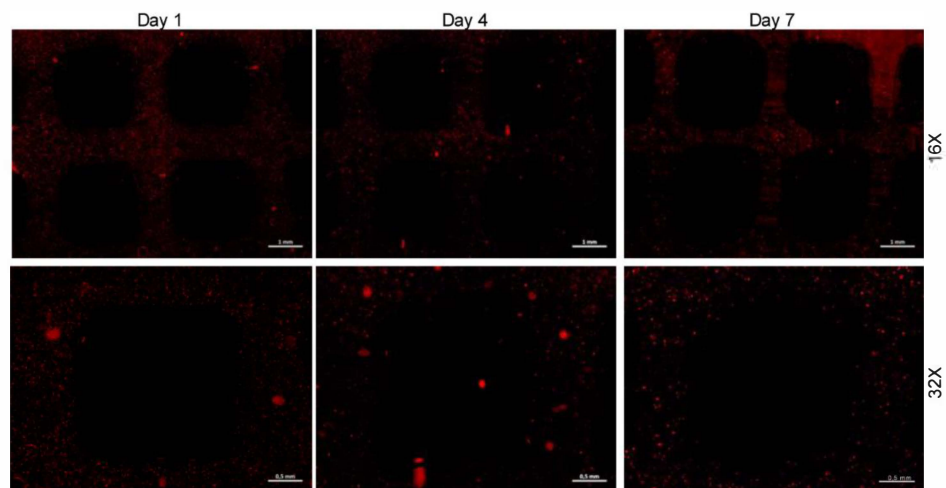

Supplementary Figure S2

A

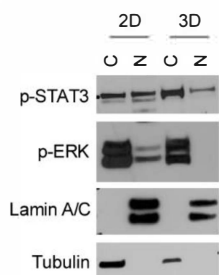

B

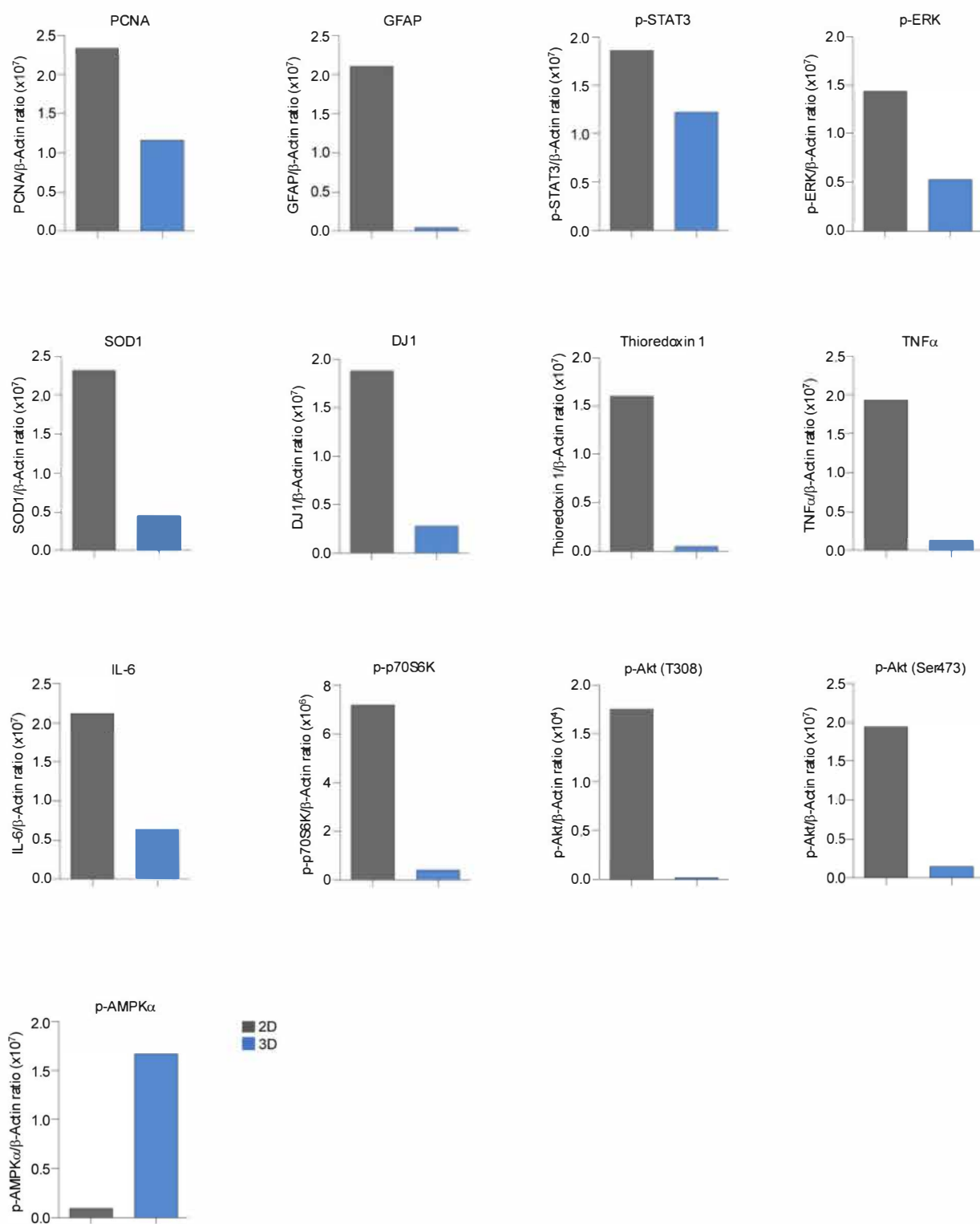

Supplement: Supplementary file 1 [file genes-15-01414-s001.zip › genes-3267825-supplementary.pdf]
